# Switching health insurers: the role of price, quality and consumer information search

**DOI:** 10.1007/s10198-015-0681-1

**Published:** 2015-03-28

**Authors:** Lieke H. H. M. Boonen, Trea Laske-Aldershof, Frederik T. Schut

**Affiliations:** Institute of Health Policy and Management, Erasmus University Rotterdam, PO Box 1738, 3000 DR Rotterdam, The Netherlands

**Keywords:** Consumer quality ratings, Health plan choice, Information search, Managed competition, I11, I13, I18, D12, G22

## Abstract

We examine the impact of price, service quality and information search on people’s propensity to switch health insurers in the competitive Dutch health insurance market. Using panel data from annual household surveys and data on health insurers’ premiums and quality ratings over the period 2006–2012, we estimate a random effects logit model of people’s switching decisions. We find that switching propensities depend on health plan price and quality, and on people’s age, health, education and having supplementary or group insurance. Young people (18–35 years) are more sensitive to price, whereas older people are more sensitive to quality. Searching for health plan information has a much stronger impact on peoples’ sensitivity to price than to service quality. In addition, searching for health plan information has a stronger impact on the switching propensity of higher than lower educated people, suggesting that higher educated people make better use of available health plan information. Finally, having supplementary insurance significantly reduces older people’s switching propensity.

## Introduction

Health care reforms in various countries aim at improving the efficiency of health care delivery by enhancing consumer choice. Countries with a social health insurance system (e.g. Germany, Netherlands, Switzerland) focus on giving consumers an annual free choice of health insurer to motivate health insurers to act as a prudent buyer of health services on behalf of their enrollees [55]. These reforms are based on the theoretical model of payer-driven managed competition [23]. By contrast, countries with a national health service (e.g. England) typically focus on enhancing patient choice of provider by encouraging patient-driven rather than payer-driven competition [27]. In the US, the dramatic rise of high deductible, “consumer-directed” health plans is an important example of the patient-driven approach [29].

Payer-driven competition reforms rely on adequate consumer choice of health insurers. Competition among health insurers can only be effective if consumers are inclined to search for better and lower-priced insurers. If consumers do not have the information or ability to make an adequate choice among health plans, or face high search or switching costs, insurers may have insufficient incentives to improve efficiency and to accommodate consumer preferences, resulting in a loss of welfare [34].^1^ Furthermore, if the ability to switch to better health plans varies across different types of consumers having specific preferences, the efficiency loss of inadequate health plan choice may vary across consumers. Since adequate health plan choice by consumers is an essential precondition for a successful performance of health care systems based on managed competition, understanding the determinants of consumer switching behaviour and the sensitivity of consumers to the price and quality of health plans is of crucial importance.

The central aim and main contribution of this paper is to improve our understanding of consumers’ switching behaviour in the context of a health insurance market with managed competition. We examine the relationship between individuals’ switching propensity and a variety of consumer, insurer and health plan characteristics. To this end, we exploit a rich set of panel data on health plan choice in the Netherlands over the period 2006–2012, including both health plan price and quality measures, and a comprehensive set of individual consumer characteristics, including age, gender, health status, education level and information on individual search behaviour. The Netherlands provides an interesting setting for investigating this, because after a major health reform in 2006 it is widely perceived as a frontrunner in implementing managed competition in health care [55].

The paper is structured as follows. In “Previous findings”, we briefly discuss the previous literature on determinants of health plan choice. The “Institutional setting” section describes the Dutch institutional setting. The “Model” section sets out the method and the “Data” section the data. Estimation results are discussed in “Results” and the “Discussion and conclusion” section concludes.

## Previous findings

Most studies about health plan choice are performed in the context of the US group health insurance market. Outside the US, most studies on health plan choice are performed in Germany, the Netherlands, and Switzerland, where individuals have a free choice of insurer during periodic open enrollment seasons. The various studies show that a variety of both consumer and health plan characteristics influence consumer choice and switching behavior.

### Health plan characteristics

Empirical studies on health plan choice in the US have all found that out-of-pocket price is an important determinant of health plan choice [7, 10, 12, 48, 53]. Many studies found that consumers are also sensitive to quality differences [3, 13, 21, 33, 36, 49, 59]. Wedig and Taj Seale [59] found that subjective quality ratings in report cards influence health plan choices and increased the price elasticity of demand for health insurance. Beaulieu [3] and Scanlon et al. [49] showed that enrollees were more likely to avoid plans with a low quality rating. Jin and Sorensen [33] found that the dissemination of quality information did have a positive effect on health plan choice and concluded that this information did not simply mirror what was already known. Dranove and Sfekas [21] showed that, when hospital report cards provide information that differs from patients’ prior beliefs, patients tended to shift away from hospitals with negative news, rather than shifting towards hospitals with positive news. Dafny and Dranove [13] found that market-based learning had a larger impact than public report cards and that the effect of those report cards was entirely driven by consumer satisfaction scores. Liu et al. [39] found that low-income parents chose health plans with higher CAHPS scores for their newly enrolled children. Generally, based on a review of the literature, Kolstad and Chernew [36] concluded that the evidence suggests that consumers tended to choose better-performing health plans and were responsive to initiatives that provided quality information. Kling et al. [35], however, found evidence of substantial “comparison friction”, that is the wedge between the availability of comparative information and consumers’ use of it. They found that actively providing personalized information was much more effective than having consumers actively access information themselves, even when the cost of acquiring information is small.

In the individual health insurance markets with managed competition in Germany, the Netherlands, and Switzerland, consumers have been found to be moderately sensitive to out-of-pocket price [5, 20, 25, 37, 51, 52, 56]. Schmitz and Ziebarth [50] showed that a radical change in the premium structure of German health insurance in 2009 resulted in a threefold increase in the price elasticity of health insurer choice and a sixfold increase in individuals’ switching probability. Their findings suggest that people are very price sensitive when the reference out-of-pocket price is set at zero, as in the case of the new German health insurance scheme. Moreover, Frank and Lamiraud [25] found that, in Swiss local health insurance markets, consumer price sensitivity declined as the number of choices grew because a large choice set makes selecting an efficient health plan more difficult and costly. Wuppermann et al. [60] found the same for retirees in Germany, who made better choices when the plan menu was smaller. So far, however, little is known about the role of quality in health plan choice in these countries.

### Consumer characteristics

Next to health plan characteristics, consumer characteristics also play an important role in the decision-making process. There is ample empirical evidence from a variety of countries that younger, male, healthier and higher educated enrollees are more willing to switch plans [32, 46, 48, 53, 56, 60]. Several studies on health plan choice by Medicare beneficiaries have found that prices matter for retirees but substantially less than for active employees [8–10]. As discussed by Buchmueller et al. [10], the exact reasons for this result are not clear, but the most likely explanations are that elderly people may have less cognitive ability to make informed choices and may have less access to informal sources of information (such as feedback from fellow employees) [43]. Studies on health plan choice in Medicare Part D providing prescription drug benefits also found that Medicare beneficiaries’ health plan choices were far from optimal and became worse as beneficiaries aged, suggesting that elderly people are often not capable of gathering sufficient information to choose the cheapest plan that meets their medication needs [1, 30, 61]. Wuppermann et al. [60] found that German retirees with lower than median education appeared less sensitive to potential savings from health plan choice than those with more education.

Research has also shown that search behaviour influences consumers’ propensity to switch. Consumers who actively search for quality information increase their capacity to make better choices and are more likely to switch plans [36]. Finally, consumers’ insurance status matters. In the Netherlands and Switzerland, consumers with comprehensive supplementary insurance are reluctant to switch plans because they expect not to be able to obtain equally comprehensive supplementary insurance from another insurer [5, 17, 22]. Since basic and supplementary coverage are usually sold as a joint product, this implies that consumers are also less inclined to switch plans for basic insurance. Furthermore, in the Netherlands, it has also been shown that having group insurance is likely to have a negative impact on consumers’ willingness to switch plans because such contracts are often concluded for several years [40].

## Institutional setting

Since the 1990s, the Dutch health care system has been in transition from supply-side government regulation towards managed competition [54]. A major step in this transition process was the introduction of the Health Insurance Act (HIA) in 2006. The HIA is based on the principles of managed competition within the context of a national health insurance system, under which all persons who legally live or work in the Netherlands are obliged to buy, on an annual basis, a legally defined comprehensive basic benefit package from a private health insurer, including primary care, medical specialist and hospital care, and prescription drugs [24]. With the introduction of the HIA, people are allowed to switch annually between health insurers. Health insurers are obliged to accept all applicants for the basic benefit package, irrespective of their individual risk profile, at a community-rated premium. In addition to the community-rated premium charged by the insurer, people have to pay an income-related contribution to a Risk Equalization Fund (REF), administered by the government. Expected differences in individual health care expenditure are equalized by means of risk-adjusted capitation payments to health insurers from this REF. Lower-income people are entitled to an income-related premium subsidy, paid by government out of general tax revenues. Before 15 November each year, health insurers are obliged to publish the terms of next year’s insurance contracts, including next year’s premiums. During a 2-month open enrollment period in December and January, people are free to choose another contract or another health insurer.

The basic idea behind the managed competition model is that consumers put pressure on health insurers to provide good service and efficient care at a reasonable price. Since the introduction of the HIA, consumer ratings of the quality of health insurers have been annually measured and publicly disseminated to facilitate consumer choice. Insurers are allowed to selectively contract with healthcare providers and may offer a variety of basic insurance products, e.g. higher-priced policies offering unrestricted provider choice and lower-priced policies offering limited provider choice (preferred provider arrangements or limited provider plans). In practice, however, health insurers hardly used the opportunity to selectively contract with providers and to offer limited provider plans [6, 44]. There is a uniform mandatory deductible that is set annually by the government, implying that all people (except children under 18) have to pay a certain amount of expenses out of pocket before the insurers start covering expenses (in 2012, this amount was €220 per year). On top of that, people may opt for a voluntary deductible at five different levels, from €100 up to €500 per year in return for a premium discount.^2^ In sum, during the study period (2006–2012), basic insurance contracts were highly standardised, primarily varying in the level of premium and service quality.

Next to individual contracts, insurers are also allowed to conclude group contracts with any legal entity (e.g. employers, unions, consumer and patient organizations). Health insurers are required to offer the same contracts to individuals and groups but are allowed to offer a group discount of up to 10 % of the individual rate. At the open enrolment season, people with a group contract are also free to choose another individual or group contract offered by another insurer. Furthermore, in addition to basic insurance, health insurers are allowed to offer supplementary insurance products for services not included in the basic benefits package, among which dental care for adults and physiotherapy are the most important. In contrast to basic insurance, supplementary insurance is not regulated by the government (except for solvency requirements). Hence, insurers are free to risk-rate premiums and to refuse applicants. Nevertheless, during our study period. most supplementary insurance contracts had community-rated premiums or a few age-rating bands [47]. Although people can buy basic and supplementary coverage from a different insurer, insurers actively discourage this by requiring high surcharges on the premium of supplementary insurance contracts. In practice, therefore, basic and supplementary insurance are sold as a joint product. About 85 % of the Dutch population has some supplementary insurance coverage [44].

## Model

In line with other econometric models of health plan choice, we model individuals’ switching propensity as a function of health plan and consumer characteristics [2]. We used the empirical findings on the determinants of health plan choice (see “Previous findings” section) and the specific features of the Dutch managed competition setting (see “Institutional setting” section) to model individuals’ switching behaviour in Dutch health insurance market. Specifically, we examine the determinants of individuals’ switching propensity over the period 2006–2012 by using an unbalanced panel to estimate the following random effects logit model in which the decision to switch at the beginning of year *t* depends on individual characteristics and the features in year *t* of individuals’ health plan in year *t* − 1:

1 $${ \Pr }\left( {\text{Switch}} \right)_{i,t} = \, {{{ \exp }\left( {\beta_{1} X_{i,t} + \beta_{2} Z_{i,t} + \beta_{3} S_{i,t} + \gamma Z_{{i,t^{*} }} S_{i,t} } \right)} \mathord{\left/ {\vphantom {{{ \exp }\left( {\beta_{1} X_{i,t} + \beta_{2} Z_{i,t} + \beta_{3} S_{i,t} + \gamma Z_{{i,t^{*} }} S_{i,t} } \right)} {\left( { 1+ { \exp }\left( {\beta_{1} X_{i,t} + \beta_{2} Z_{i,t} + \beta_{3} S_{i,t} + \gamma Z_{{i,t^{*} }} S_{i,t} } \right)} \right)}}} \right. \kern-0pt} {\left( { 1+ { \exp }\left( {\beta_{1} X_{i,t} + \beta_{2} Z_{i,t} + \beta_{3} S_{i,t} + \gamma Z_{{i,t^{*} }} S_{i,t} } \right)} \right)}}$$

where *X*_*i,t*_ is the vector of background characteristics of individual *i* in year *t* (age, gender, level of education, self-perceived health status and insurance status), *Z*_*i,t*_ is the vector of insurer characteristics (premium and quality rating) in year *t* of individual* i*’s health insurer in year *t* *−* 1, *S*_*i,t*_ is a dummy variable indicating whether or not individual *i* in year *t* actively searched for information about health plans, and *β*_*1*_ *, β*_*2*_ *, β*_*3*_, and *γ* are the associated vectors of coefficients.^3^

The dependent variable in our model is the decision of the respondent to switch health insurer or not. Switching is defined as switching from one health insurer to another. In the Dutch health insurance market, several large health insurance companies offer policies under different labels (i.e. separate legal entities operating under a different brand name). Consumers who switch between different labels from the same health insurance company are also identified as switchers. In addition, most health insurers (labels) offer several slightly different basic health insurance policies (typically a policy offering service benefits and a policy offering indemnity or cash payments). People opting for another policy offered by the same insurer are not considered as switchers in our analysis.

For the independent variables, we have two groups. The first group consists of the consumer characteristics (*X*), including age, gender, education level, and health status. In line with previous findings, we expect that the propensity to switch will be negatively related to age, and positively related to health status and education level. A higher level of education is likely to be associated with higher cognitive skills to compare health plans and therefore with lower transition costs. This association is supported by several studies [38, 60]. We also included two variables indicating whether or not the respondent participated in a group contract or had a supplementary insurance in year *t* − 1. Both variables are expected to be negatively correlated with the propensity to switching in year *t*. Having supplementary insurance may reduce people’s switching propensity because of the complexity of supplementary insurance contracts and because people may fear not to be accepted by another insurer at the same conditions. The latter may be particularly true for the elderly and chronically ill [22, 47]. Hence, in addition to having less cognitive ability to make informed choices, the lower switching propensity of elderly people may also be caused by fear of being rejected for supplementary insurance. Participating in a group contract may reduce people’s switching propensity because of a premium discount for the group contract or because people may simply follow the group decisions.

The second group of independent variables consists of insurer characteristics. As explained above, during the study period, health insurers primarily distinguished themselves by differences in premium and service quality, since co-payments (i.e. deductibles) are standardised and selective contracting was almost absent. Since we aim to measure the effect of premium and quality on switching propensity, we include the information on premiums and quality ratings that is available to consumers during the 2-month open enrollment period (December year *t* − 1, January year *t*) as independent variables. What matters is how the price and quality of an individual’s health plan compares to other similar alternatives. Since the benefits package is standardised and insurers contract with almost all providers (so consumers can switch insurers without having to switch providers), all available health plans belong to people’s relevant comparison group. Each year, people can choose among all available health plans, and for this reason we included all health plan prices and quality ratings in year *t* in our estimated model.

The premium variable in our model is the premium for basic health insurance charged in year *t* by people’s incumbent insurer in year *t* – 1.^4^ By the end of year *t* − 1, people can compare all health plan prices in the coming year (year *t*) since these prices have ultimately to be announced by insurers by November 15. Since health plan premiums usually change each year, we expect that at least some people compare these prices and may decide that next year’s premium of their current health insurer relative to the others makes it worth switching. Hence, we expect that the premium asked in the next year (*t*) by the insurer chosen by an individual in year *t* − 1 is positively related to that individual’s propensity to switch in year *t*.^5^

The quality variable we included is the rating of people’s current insurer (in year *t* − 1) available at the public website during the open enrollment period. This quality score is based on consumer ratings of different aspects of insurers’ service quality that are annually collected through a standardised and validated survey among a representative group of the insured population (see “Data” section). Although these quality ratings are measured by a survey among consumers in year *t* − 1, we have labelled this variable as quality rating in year *t* because this information is only available when people have to choose for a health insurer in year *t*. We expect that, if this quality rating adequately reflects consumers’ perception and experience of the service quality of their insurer, people with a low-rated insurer are more likely to switch than people with a high-rated insurer.^6^

We are specifically interested in those who actively search for health plan information because we expect that these individuals are more inclined to switch. Therefore, in the survey, we asked people whether they actively searched for information before deciding to switch (or not).^7^ To investigate how people react to consumer information, we interacted health plan premiums and quality ratings with a dummy variable (*S*) indicating whether the individual actively searched for health plan information. Since Kolstad and Chernew [36] found that consumers who actively search for health plan information increased their capacity to make better choices and were more likely to switch plans, we expected that people who actively search for information are more sensitive to price and quality. We also investigated whether higher educated people who actively search for information are more likely to switch than lower educated people, by interacting these variables.^8^ For instance, Lave et al. [38] found that higher educated employees appeared to be more willing to examine the benefits and costs of specific health plans in the US. If information search has a stronger impact on the switching propensity of higher educated people, this may indicate that higher educated people make more effective use of the available health plan information.

In addition to mandatory basic insurance, about 85 % of the Dutch population also voluntarily buys supplementary insurance. Since supplementary insurance products are highly differentiated, premiums are hard to compare and we were not able to include a reliable price variable for supplementary insurance. Van Dijk et al. [56], however, found that the price elasticity of supplementary insurance for the most common benefit package was not significant different from zero. They argued that this was likely due to the enormous product differentiation making price comparisons almost impossible. Frank and Lamiraud [25] and Wuppermann et al. [60] found that, as the number of health plan choices grew consumer price sensitivity declined. Based on these findings, we expected that omitting the price of supplementary insurance may not have a strong impact on the estimation results.

Finally, we included year-dummies to correct for year-specific differences. In particular, 2006 was a special year because of the introduction of the Health Insurance Act, which made many people reconsider their previous health plan choice.

## Data

For this study, we use two different datasets. The first dataset is constructed using the data of annual surveys about health plan choice from 2006 to 2012. For each year, descriptive statistics are summarised in Table 1. Since the introduction of the new Health Insurance Act in 2006, surveys were sent out each year to a panel of about 2,000 respondents. Our sample is an unbalanced panel: 19.6 % of the respondents participated in each of the 6 years, 31.1 % in only 1 year and the remaining respondents in 2–5 years. On average, respondents are present for 3.2 years in the sample. Each year, the survey was sent out in February, immediately after the 2-month open enrollment period (December and January). All respondents participated in an internet-based household panel (http://www.centerdata.nl). Compared to the Dutch population, our sample is fairly representative with respect to age, is composed of slightly fewer respondents with a bad or mediocre self-reported health status and is slightly more highly educated [11].

**Table 1:** Descriptive statistics of survey respondents

	2006	2007	2008	2009	2010	2011	2012
	% of total	% of total	% of total	% of total	% of total	% of total	% of total
	*n* = 2147^a^	*n* = 2094^a^	*n* = 1681^a^	*n* = 1725^a^	*n* = 1967^a^	*n* = 1937^a^	*n* = 1838^a^
Switching rate							
% Switched	26.2	4.5	2.9	3.0	3.9	4.0	4.7
Use of information							
Searched for health plan information	93.7	37.5	17.3	35.0	40.3	43.3	45.7
Type of insurance							
Group contract	55.6	61.1	66.4	67.7	69.2	70.8	71.6
Individual contract	44.4	38.9	33.6	32.3	30.8	29.2	28.4
Insurance policy							
Only basic benefit package	7.0	7.5	8.1	9.3	9.3	8.5	12.1
Supplementary insurance	93.0	92.5	91.9	90.7	90.7	91.5	87.9
Gender							
Male	52.1	52.2	53.5	54.8	53.9	53.8	54.0
Female	47.9	47.9	46.5	45.2	46.1	46.2	46.0
Age							
18–35 years	24.5	24.2	18.4	16.6	15.7	12.2	9.1
36–50 years	29.3	28.5	25.8	25.3	26.2	25.9	25.5
51–64 years	27.4	28.9	32.4	32.6	34.0	35.1	34.4
65 years and older	18.9	18.4	23.4	25.3	24.2	26.9	31.0
Average age male	51.2	51.8	54.0	54.7	54.5	56.5	57.3
Average age female	46.6	46.3	49.8	51.1	51.2	52.4	54.2
Education							
Low and intermediate level	64.6	65.0	65.6	63.2	61.7	60.9	61.3
High level	35.4	35.0	34.4	36.8	38.3	39.1	38.7
Self-reported health status							
Bad/mediocre	14.0	14.4	14.9	16.5	15.6	16.3	16.5
Good	58.1	55.4	57.4	55.5	54.9	56.3	53.4
Very good/excellent	27.9	30.2	27.7	28.0	29.5	27.4	30.1
Premium/quality rating							
Average monthly premium (SD)	88.3 (2.7)	95.3 (1.4)	91.2 (2.2)	92.9 (3.1)	96.9 (2.7)	103.3 (2.9)	107.6 (3.3)
Average quality rating (SD)	11.7 (3.1)	10.8 (3.3)	10.7 (2.8)	11.3 (2.3)	10.7 (3.1)	11.1 (2.6)	11.2 (2.5)

^a^ *n* represents the sample size per year. For a few respondents, (some) background characteristics are missing

As shown in Table 1, the percentage of switchers was extremely high (26 %) after the introduction of the new Health Insurance Act in 2006, and dropped to about 4 % in subsequent years. Similarly, the percentage of respondents searching for information about health insurance dropped sharply from more than 90 % in 2006 to about 40 % in the following years. The obvious reason for this is that the reform of the health insurance market urged people to reconsider their previous health plan choice because all health insurance products changed substantially.^9^

The second dataset is derived from the government website http://www.kiesbeter.nl which publishes comparative information about price and coverage of health insurance products and about consumer quality ratings of health plans. For each health insurer the price of the basic benefit package is available on this website. As shown in Table 1, the average monthly out-of-pocket premium per adult increased from about €88 in 2006 to about €108 in 2012. The drop in average premium in 2008 can be explained by the introduction of a mandatory deductible instead of a no-claim rebate.

Quality ratings are based on a standardized and validated method to measure different aspects of insurers’ quality from a consumer perspective [16, 18]. Each year, a representative sample of enrolees of each health insurer fills in a survey with questions how they value their insurer’s products and performance [4, 14, 15, 18, 19, 31, 45]. Consumer ratings pertain to two dimensions of insurers’ performance. The first dimension includes various aspects of the service quality provided by the insurer. Six service items are distinguished: personal approach, provision of information, telephone responsiveness and assistance by the service desk, completing bills, and the comprehensibility of the levels of copayments. The second dimension includes a rating of the health services quality contracted by health insurers. Consumer ratings with regard to this second dimension of performance hardly differed across insurers during the study period. Only about 4 of the 31 insurers scored significantly differently from the average. One obvious reason for this is that during this period health insurers hardly engaged in selective contracting of health care providers [6], resulting in unrestricted provider choice for consumers irrespective of their choice of health plan. Due to the lack of variation in consumer ratings with regard to second dimension of insurers’ performance (contracted health care quality), we focused in our analyses exclusively on consumer ratings with regard to the first dimension (service quality).

On the website, each of the rated service quality items is presented by a ‘star’ score, indicating whether the insurer scores below average (1 star), average (2 stars) or above average (3 stars). Similar to empirical studies on health plan choice in the US [3, 39], we constructed an aggregated quality rating based on the scores on the six individual items. The aggregated quality rating varies between 6 (all items below average) and 18 (all items above average). We used the total number of stars for each insurer as a quality variable. In addition to the star scores, the website also publishes a general consumer score of health insurers. Since the aggregated quality rating gives more differentiated insight in the perceived quality difference and displays a larger variation than the general score, we included only the aggregated quality rating in our analysis. Table 1 shows the mean and standard deviation of the aggregated quality rating (number of stars). On average enrollees have an insurance policy with 11 stars. The variation between insurers decreased slightly over the years, indicating that differences in quality ratings between insurers became smaller.

Interestingly, we did not find any correlation between an insurer’s out-of-pocket premium and quality rating. In each year of our study period, the correlation coefficient is very low (on average 0.08, ranging from 0.00 to 0.25) and not statistically significant different from zero. This implies that people do not need to make a trade-off between price and service quality when choosing a health insurer.

## Results

Table 2 presents the results of our regression. Since the coefficients of logistic regressions give no information on the magnitude of the impact of the explanatory variables on the decision whether or not to switch, we also present the marginal effects to facilitate the interpretation of our findings.

**Table 2:** Estimated coefficients and marginal effects (in  %-points) of the determinants of the health plan switching over the period 2006–2012

	Coefficients	Marginal effects^a^
Consumer characteristics		
Age (in years)^b^	−0.03***	−1.1
Female	0.07	0.31
High education (compared to low/intermediate)	−0.27	1.65
Good health (compared to mediocre/bad health)	0.23	0.98
Excellent health (compared to good health)	0.50***	2.27
Supplementary insurance	−0.34***	−1.56
Group contract	−0.52***	−2.23
Searched for health plan information	−3.53*	6.11
Insurer characteristics		
Monthly premium offer (in *t*) by insurer (in *t* − 1) (in euros)	0.02	2.78
Quality rating (in *t*) of insurer (in *t* − 1)	−0.11***	−0.36
Interaction effects		
Education × searched for information	0.73***	(See Table 4)
Premium × searched for information	0.05***	(See Table 4)
Quality rating × searched for information	0.03	(See Table 4)
Year dummies		
Year 2007	−2.24***	−6.64
Year 2008	−2.08***	−5.46
Year 2009	−2.25***	−6.02
Year 2010	−2.33***	−6.53
Year 2011	−2.59***	−7.34
Year 2012	−2.78***	−7.61
Constant	−1.39	
Number of observations	11,598	
Number of groups	3570	
Baseline switching rate	5.44 %	
Intraclass correlation (ICC)	0.059	

* p < 0.10; ** *p* < 0.05; *** p < 0.01

^a^The marginal effects for dummy variables are expressed as the discrete change from the base level (in %-points). For continuous variables, we estimated the average marginal effect for a one unit change in the independent variable (see, for example, [41, 42]). Since an average individual does not exist, we computed the marginal effects as the mean of the marginal effects over each individual [26, 53]

^b^For age, we estimated the average marginal effect for a 10-unit change (10-year intervals) change in the independent variable age

Our findings on the impact of consumer characteristics on health plan switching are in line with those of previous studies. As expected, the probability to switch decreases with age. On average, over the period 2006–2012, a 10-year increase in age leads to a 1.1 %-point decrease in switching probability relative to a base rate of 5.4 %. To examine whether switching determinants differ for different age groups, we have also split the sample into four broad age categories. The results are presented in Table 3. As shown in Table 3, the switching propensity decreases from about 10 % for the lowest age group (18–35) to about 1.4 % for the highest age group (65+).^10^

**Table 3:** Estimated coefficients and marginal effects (*ME*) of the determinants of the health plan switching over the period 2006-2012 for different age groups

Age groups	18–35		36–50		51–64		65+	
	Coeff	ME	Coeff	ME	Coeff	ME	Coeff	ME
Consumer characteristics								
Female	−0.01	−0.06	0.21	1.19	0.24	0.91	−0.58	−0.67
High education	−1.05**	2.92	−0.44	1.98	0.60	1.67	−1.12	0.28
Good health	0.66	4.53	0.20	1.11	0.12	0.44	0.36	0.43
Excellent health	1.04**	7.23	0.49	2.94	0.26	1.01	0.84	1.30
Supplementary insurance	−0.02	−0.15	−0.16	−0.95	−0.50**	−2.13	−0.88**	−1.31
Group contract	−0.67***	−4.44	−0.39**	−2.21	−0.44**	−1.67	−0.98**	−1.12
Searched for information	−8.07**	9.06	−1.99	8.48	0.30	5.54	−3.70	1.79
Insurer characteristics								
Monthly premium	−0.0	4.49	0.05	4.86	0.02	1.03	0.10	2.35
Quality rating	−0.08	−0.57	−0.04	−0.42	−0.20**	−0.37	−0.41*	−0.12
Interaction effects								
Education × info search	1.64***	^a^	0.89*	^a^	−0.20	^a^	1.38	^a^
Premium × info search	0.10**	^a^	0.04	^a^	0.01	^a^	0.03	^a^
Quality rating × info search	−0.01	^a^	−0.05	^a^	0.11	^a^	0.31	^a^
Year dummies								
Year 2008	−2.87***	−14.00	−2.37***	−8.97	−1.58***	−4.34	−2.21***	−1.80
Year 2009	−2.28***	−10.28	−2.24***	−7.33	−1.73***	−4.15	−2.77***	−1.54
Year 2010	−2.95***	−11.61	−1.58***	−6.59	−2.13***	−4.85	−4.50***	−2.03
Year 2011	−2.56***	−11.27	−2.49***	−8.81	−1.81***	−4.90	−3.94***	−2.61
Year 2012	−2.50***	−10.90	−3.07***	−10.87	−2.19***	−5.63	−3.50***	−3.44
Constant	0.62		−6.48		−2.77		−8.51	
Number of observations	2057		3039		3743		2759	
Number of groups	877		1144		1237		879	
Baseline switching rate	9.95 *%*		7.18 *%*		4.47 *%*		1,41 *%*	

* *p* < 0.10; ** p < 0.05; *** p < 0.01

^a^Marginal effects of the interaction effects at the various levels (see Table 4) are available from the authors upon request

The results presented in Table 2 show that, relative to the base rate, higher educated people have a 1.7 %-point higher propensity to switch than those with an intermediate and low level of education. From Table 3, it follows that education is a primarily important determinant of switching for the two lowest age groups. In addition, Table 2 shows that people with a very good/excellent health status have a 2.3 %-point higher chance to switch than people in good health, who in turn have a 1.0 %-point higher probability to switch than people with a bad or mediocre health status. As shown in Table 3, the impact of health on switching is most prominent within the youngest age group. Furthermore, having supplementary insurance and participating in a group contract both have a significant negative impact on people’s propensity to switch health plans (by 1.6 and 2.2 %-points; see Table 2). Table 3 shows that the negative effect of supplementary insurance on switching particularly holds for the two highest age groups. This corroborates findings from earlier studies based on questionnaires that older and unhealthy people more often do not contemplate switching because they fear not to be accepted for supplementary insurance [47, 22]. Not surprisingly, people who indicated that they actively searched for information about health plans are much more likely to switch (6.1 %-points). Table 3 shows that this particularly holds for the lowest age group.^11^

Our results on the impact of insurer characteristics on health plan switching indicate that consumers base their switching decisions both on price and service quality. As expected, premium has a significant positive effect on the propensity to switch. Enrollees of an insurer charging a relatively high premium are more inclined to switch than those who are charged a relatively low premium. As shown in Table 2, a 10 % higher than average monthly premium (equivalent to about €10), raises the propensity to switch with 2.8 %-point relative to the base rate of 5.4 %. In addition, the results show that the estimated switching propensity increases from less than 3 % at a monthly out-of-pocket premium of €80 to more than 11 % at monthly premium of €110.

We find that consumers are also sensitive to insurers’ service quality as measured by the aggregated quality rating. As shown in Table 2, the results demonstrate that enrollees having an insurer with a lower quality rating are more inclined to switch than enrollees insured with an insurer with a high quality rating. A one star higher than average quality rating, reduces the propensity to switch with 0.4 %-points relative to the 5.4 % base rate.

Table 3 shows that sensitivity for price and quality differs across age groups. People in the youngest age group (18–35 years) appear to be more sensitive to price, whereas the people in other age groups appear to be more sensitive to quality.

We also find that searching for health plan information has a stronger impact on peoples’ sensitivity to price than to service quality. As shown in Table 2, the interaction between premium and information search is positive and statistically significant, implying that those who actively search for information are more inclined to switch when their insurer charges a high premium next year than those who do not search for information. As shown in Table 3, this is particularly the case for people in the lowest age group. By contrast, the interaction between quality rating and information search is not statistically significant.

**Fig. 1:**
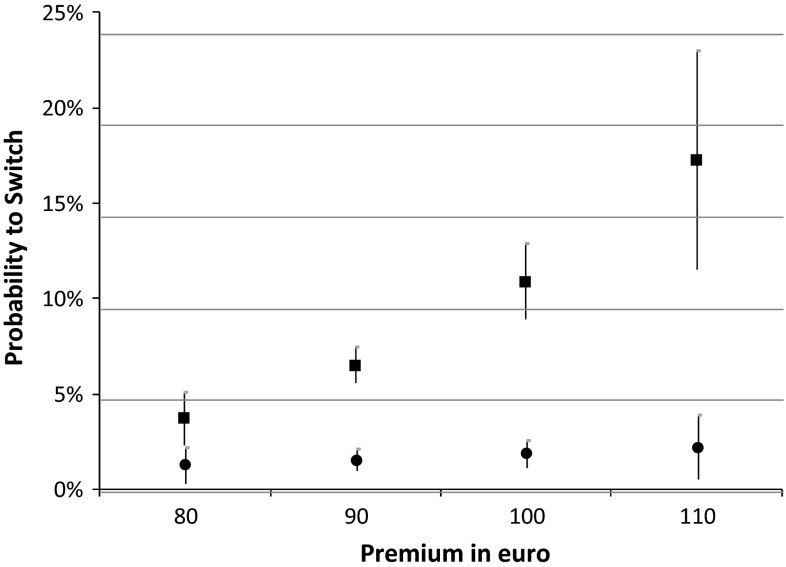
Estimated switching propensities at different premium levels for people who searched for information and for people who did not, over the period 2006−2012. The *squares* indicate the switching propensity of those who searched for information, and the *circles* the switching propensity of those who did not search for information. The *vertical lines* indicate the 95 % confidence intervals

Table 4 shows that the switching propensity of those who searched for information significantly increases with price, from 3.7 % at the lowest out-of-pocket premium (€80) to 17.2 % at the highest out-of-pocket premium (€110). By contrast, for those who did not search for information the switching propensity hardly increases with price, from 1.5 % at the lowest premium to only 2.4 % at the highest premium, and this difference is not statistically significant. As a consequence, the marginal effect of information search at different premium levels (i.e. the difference in switching propensities between both groups) increases from 2.2 %-points at a monthly premium of €80 to 14.8 %-points at a monthly premium of €110 (Table 4, last column). This implies that switchers who search for information are more sensitive to price than those who switch but did not search. This is illustrated by Fig. 1, which shows that at higher premium levels switching propensities for those who search for information significantly increase, whereas switching propensities for those who do not search are fairly constant.

**Table 4:** Marginal effects (*ME*) of information search at different levels of education, premium and service quality over the period 2006−2012 (in %-points)

	Switching propensity in % (95 % CI)		ME of information search (in % points)
	Searched for information	No information searched	
Education × searched for information			
Low/intermediate education	6.83 (5.78; 7.87)	1.91 (1.31; 2.52)	4.92
High education	9.86 (8.30; 11.42)	1.48 (0.84; 2.13)	8.38
Premium × searched for information			
€80	3.68 (2.31; 5.05)	1.45 (0.49; 2.41)	2.23
€90	6.49 (5.57; 7.41)	1.73 (1.17; 2.28)	4.76
€100	10.86 (8.88; 12.84)	2.05 (1.30; 2.80)	8.81
€110	17.23 (11.52; 22.94)	2.43 (0.71; 4.15)	14.80
Quality rating × searched for information			
6 stars	10.94 (8.97; 12.91)	2.89 (1.65; 4.13)	8.05
7 stars	10.24 (8.58; 11.90)	2.61 (1.62; 3.61)	7.63
8 stars	9.57 (8.17; 10.98)	2.36 (1.56; 3.17)	7.21
9 stars	8.95 (7.74; 10.15)	2.14 (1.47; 2.80)	6.81
10 stars	8.35 (7.27; 9.43)	1.93 (1.36; 2.49)	6.42
11 stars	7.79 (6.78; 8.80)	1.74 (1.22; 2.25)	6.05
12 stars	7.26 (6.26; 8.26)	1.57 (1.07; 2.07)	5.69
13 stars	6.76 (5.74; 7.79)	1.41 (0.91; 1.91)	5.35
14 stars	6.30 (5.22; 7.38)	1.27 (0.76; 1.78)	5.03
15 stars	5.86 (4.71; 7.00)	1.14 (0.61; 1.67)	4.72
16 stars	5.44 (4.24; 6.65)	1.03 (0.49; 1.57)	4.41
17 stars	5.06 (3.79; 6.32)	0.92 (0.37; 1.47)	4.14
18 stars	4.69 (3.38; 6.01)	0.83 (0.28; 1.38)	3.86

**Fig. 2:**
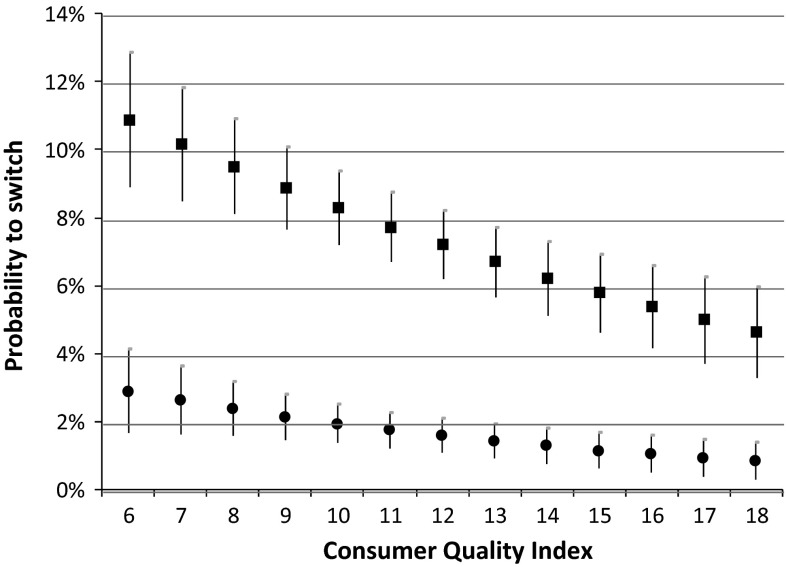
Estimated switching propensities at different quality ratings for people who searched for information and for people who did not, over the period 2006–2012. The *squares* indicate the switching propensity of those who searched for information, and the *circles* the switching propensity of those who did not search for information. The *vertical lines* indicate the 95 % confidence intervals

The results in Table 4 (last column) also show that the marginal effect of information search is higher at lower quality levels, ranging from 3.9 %-point at the highest quality rating (18 stars) to 8.1 %-point at the lowest quality rating (6 stars). This suggests that switchers who search for information are more sensitive to quality than switchers who do not search. However, since the confidence intervals overlap for differences in quality up to 5 stars, only large differences in quality have a distinct impact on the switching propensity of people who actively search for information. The relationship between insurer quality ratings and the switching propensities for both groups is illustrated by Fig. 2.

Figure 2 shows that, at higher quality ratings (the number of stars), the propensity to switch declines at a higher rate for those who search than for those who do not, but that, for small changes in quality, confidence intervals overlap.

The results presented in Table 4 also show that higher educated people who searched for information have a 3 %-point higher (9.9 vs. 6.8 %) switching propensity than lower educated people who search for information. By contrast, when people do not search for information, switching rates do not differ between higher and lower educated people. This finding suggests that higher educated people make more effective use from the available information than lower educated people.

Finally, as expected, all year dummies relative to 2006 were large and highly significant, reflecting the all-time high switching rate at the start of the reforms. Since the context in 2006 differs much from that in 2007 to 2012, we also tested whether a model over the time period 2007–2012 led to other results. The estimation results for the period 2007–2012 are included in the “Appendix”. The results show that leaving out 2006 does not substantially alter the results. Except for the variable excellent health, both the sign and the magnitude of the estimated coefficients are quite similar.^12^

This finding suggests that the introduction of the reforms merely had an impact on the switching rate but not on its determinants.

## Discussion and conclusion

We find that consumers in the competitive Dutch health insurance market base their switching decisions not only on price but also on service quality. Although service quality matters, publicly available quality information seems to play a limited role in motivating consumers to switch. This is because we find that searching for health plan information significantly increases peoples’ sensitivity to price but not to quality. Only large differences in quality (exceeding five stars) seem to have a distinct impact on the switching propensity of people who actively search for health plan information. This suggests that published quality ratings may be of limited use when people decide to switch because they may know the service quality of their insurer from experience. Public investments in collecting and disseminating comparative information about health insurers’ service quality may still be worthwhile, however, because it may improve people’s switching decisions by providing useful information about the quality of other insurers. In addition, when health insurers engage in selective contracting and impose restrictions on provider choice—as they have gradually started to do in the Netherlands since 2012—the need for comparative information about the performance of health insurers—especially about the quality of the contracted provider network—is likely to increase.

We also find support of results in previous studies that the propensity to switch insurers decreases with age, and increases with education and health status. This may provide insurers with incentives to focus on accommodating the preferences of young, well-educated and healthy people. We find that young people are more sensitive to price, whereas older people are more sensitive to quality. Given that young people are more willing or able to switch, this preference heterogeneity may be a problem in a managed competition setting, since insurers may not have sufficient incentives to invest in high-quality care for the elderly and chronically ill. In addition, we find that searching for information has a stronger impact on the switching propensity of higher than lower educated people, suggesting that higher educated people use available health plan information more effectively than lower educated people.

Finally, our results also support earlier findings that older people having supplementary insurance are less likely to switch health insurers. As far as this is caused by negative spillover effects from supplementary insurance (e.g. more stringent underwriting practices) on consumer choice in basic health insurance, this may reduce the effectiveness of managed competition in health care.

A limitation of our research is that it focuses on the determinants of people’s switching propensity but does not evaluate the actual choices people made. For instance, does switching improve people’s health plan choice in terms of price and quality and how are people making a trade-off between price and (service) quality? Interestingly, making such a trade-off might not have been necessary in the Dutch health insurance market during our study period, since we found no correlation between an insurer’s price and quality rating. Another important question for further research is whether people who actively search for information make better choices in terms of price and quality, and what sources of information they actually use. Recent empirical findings in the US health insurance market show that many people do not effectively use the available health plan information [35]. An answer to these questions may provide an indication of the effectiveness of the available consumer information about health plans.

## Appendix: estimation results 2007–2012

See Tables 5 and 6.

**Table 5:** Estimated coefficients and marginal effects (in %-points) of the determinants of the health plan switching over the period 2007−2012

	Coefficients	Marginal effects^a^
Consumer characteristics		
Age (in years)^b^	−0.03***	−0.62
Female	0.05	0.10
High education (compared to low/intermediate)	−0.22	0.53
Good health (compared to mediocre/bad health)	0.06	1.27
Excellent health (compared to good health)	0.18	0.35
Supplementary insurance	−0.46**	−1.05
Group contract	−0.64***	−1.34
Searched for health plan information	−3.21	3.86
Insurer characteristics		
Monthly premium offer (in *t*) by insurer (in *t* − 1) (in euro’s)	0.01	0.80
Quality rating (in *t*) of insurer (in *t* − 1)	−0.12***	−0.13
Interaction effects		
Education × searched for information	0.60**	(Table 6)
Premium × searched for information	0.04*	(Table 6)
Quality rating × searched for information	0.06	(Table 6)
Year dummies		
Year 2008	0.12	0.24
Year 2009	−0.04	−0.08
Year 2010	−0.04	−0.07
Year 2011	−0.20	−0.38
Year 2012	−0.34	−0.61
Constant	−2.37	
Number of observations	9972	
Number of groups	3336	
Baseline switching rate	2.08 %	
Intraclass correlation (ICC)	0.087	

** p* < 0.10; ** *p* < 0.05; *** *p* < 0.01

^a^The marginal effects for dummy variables are expressed as the discrete change from the base level (in %-points). For continuous variables, we estimated the average marginal effect for a one-unit change in the independent variable (see, for example, [41, 42]). Since an average individual does not exist, we computed the marginal effects as the mean of the marginal effects over each individual [26, 53]

^b^For age, we estimated the average marginal effect for a ten unit change (10 year intervals) change in the independent variable age

**Table 6:** Marginal effects (*ME*) of information search at different levels of education, premium and service quality over the period 2007−2012 (in  %-points)

	Switching propensity in % (95 % CI)		ME of information search (in % points)
	Searched for information	No information searched	
Education × searched for information			
Low/intermediate education	3.88 (2.74; 5.01)	0.67 (0.39; 0.95)	3.21
High education	5.52 (3.87; 7.18)	0.54 (0.26; 0.82)	4.99
Premium × searched for information			
€80	1.79 (0.31; 3.28)	0.53 (−0.01; 1.07)	1.26
€90	3.03 (1.72; 4.33)	0.58 (0.27; 0.90)	2.44
€100	5.04 (3.64; 6.44)	0.64 (0.38; 0.91)	4.40
€110	8.22 (3.76; 12.68)	0.70 (0.13; 1.28)	7.51
Quality rating × searched for information			
6 stars	5.74 (3.79; 7.70)	1.06 (0.50; 1.62)	4.68
7 stars	5.45 (3.76; 7.15)	0.94 (0.50; 1.39)	4.51
8 stars	5.18 (3.69; 6.66)	0.84 (0.48; 1.20)	4.34
9 stars	4.91 (3.59; 6.24)	0.75 (0.45; 1.04)	4.17
10 stars	4.66 (3.45; 5.88)	0.66 (0.41; 0.92)	4.00
11 stars	4.43 (3.27; 5.58)	0.59 (0.36; 0.82)	3.83
12 stars	4.20 (3.06; 5.33)	0.52 (0.31; 0.74)	3.67
13 stars	3.98 (2.83; 5.13)	0.47 (0.25; 0.68)	3.51
14 stars	3.78 (2.59; 4.96)	0.41 (0.20; 0.63)	3.36
15 stars	3.58 (2.34; 4.82)	0.37 (0.16; 0.58)	3.21
16 stars	3.39 (2.10; 4.69)	0.33 (0.12; 0.54)	3.07
17 stars	3.22 (1.86; 4.58)	0.29 (0.08; 0.50)	2.93
18 stars	3.05 (1.63; 4.47)	0.26 (0.05; 0.46)	2.79

## Footnotes

1 However, as shown by Handel [28], the welfare effects of improving health plan choice by consumers are theoretically ambiguous because it may also exacerbate adverse selection. Whether improving health plan choice is welfare increasing or decreasing depends on the market environment. In the Dutch health insurance market, the potential for adverse selection is limited because insurance is mandatory for all citizens, the benefits package is standardized and insurers receive a risk-adjusted capitation payment in addition to a community rated premium (see “Institutional setting” section). The Dutch risk adjustment scheme is quite sophisticated and has been substantially improved over the last decade [57]. As explained by Handel [28], in the case of adequate risk adjustment adverse selection is effectively constrained, leaving only the positive effects of improving individual-level choices. Hence, in the Netherlands, the positive welfare effects of improving health plan choice are likely to outweigh the negative effects.

2 In 2012, only 7 % of the insured population opted for a voluntary deductible, of which about 50 % chose the highest level (€500) and about 20 % the lowest level (€100) [58].

3 All models are estimated using STATA 12.0.

4 As mentioned before, most health insurers offer several slightly different basic insurance policies (typically a policy offering service benefits and a policy offering indemnity or cash payments). Since we have no data on the actual policy chosen by the respondents, we use the (unweighted) average premium in cases of more than one policy. In addition, we also estimated the model including the lowest instead of the average premium, but this had no significant impact on the results.

5 In addition to differences in premium levels across health plans, health plan switching may also be triggered by a change in price of people’s incumbent insurer (an awareness effect). We therefore also estimated the effect of a change in price from year *t* − 1 to year *t* of people’s insurer in year *t* − 1. Since this effect was not significant and did not improve or alter the estimation results, we did not include this price variable in the estimated model presented in this paper.

6 We also examined several interaction effects. For instance, by interacting age and health with quality ratings, we examined whether older or unhealthy enrolees were more sensitive to quality and thus more likely to switch if their insurer has a low quality rating. Since none of these interaction effects were significant., we did not include the interaction variables in the estimated model presented in this paper.

7 Notice that we cannot infer from this variable whether seeking information as such has an impact on switching since people who start searching for information may be more inclined to switch anyway.

8 This interaction is included in the estimated model, but, for the sake of simplicity, was not included in the model specification.

9 Notice, however, that people were not forced to make an active health plan choice. If they did not make an active choice, they were automatically enrolled in the default (i.e. most comparable) health plan issued by their current insurer.

10 In the Netherlands, premium payments start at the age of 18 (until that age, children do not have to pay a premium because their health care expenses are paid out of general taxation). Due to this provision, price sensitivity in this group may be particularly high during their first open enrollment period. Indeed, we find that the proportion of switchers is the highest in this particular age group. Since the number of 18-year-olds in our example is small (comprising only 2.3 % of the total number of switchers), this does not substantially bias our results.

11 Notice that, because of the interaction effects, the net impact of information search on switching is positive.

12 Although the marginal effects of the model including 2006 are larger, this is mainly due to a difference in base level. Proportional to the base level, the marginal effects are quite similar.
